# Duplicate, diversify, repeat: The evolution of *NOTCH2NL*

**DOI:** 10.1016/j.xgen.2026.101274

**Published:** 2026-06-10

**Authors:** Nachshon Egyes, David Gokhman

**Affiliations:** 1 Department of Molecular Genetics, Weizmann Institute of Science, Rehovot 7610001, Israel

## Abstract

Segmental duplications are key drivers of evolutionary innovation but are also particularly challenging to study. In this issue, Real et al. resolve the genetic diversity, structural history, and regulatory landscape of the human-specific *NOTCH2NL* gene family, a likely contributor to human cortical brain expansion.

## Main text

The expansion of the cerebral cortex is among the most distinctive features of human evolution, yet the genetic changes that enabled it—and the selective pressures that fixed them—remain poorly understood. Comparative genomics has increasingly implicated structural variation, and in particular segmental duplications, as a key substrate for the emergence of lineage-specific paralogs and gene regulation.^1^ However, the forces driving and shaping these duplications, and their functional consequences, remain unclear.

**Figure 1:**
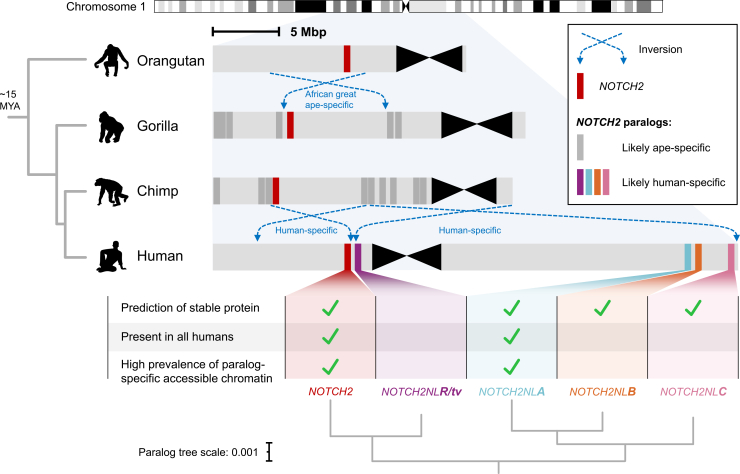
Evolutionary history and functional analysis of the *NOTCH2NL* gene family in great apes Paralog tree scale bar indicates 0.001 substitutions per site, as inferred by Real et al.^4^ using 21 kbp from intron 2. MYA, million years ago.

A compelling case is the *NOTCH2NL* (*NOTCH2-N-terminus-like*) family. Fiddes and colleagues,^2^ together with Suzuki and colleagues,^3^ previously showed that partial duplications of *NOTCH2* gave rise to four human-specific paralogs that interfere with Notch signaling, prolonging self-renewal of cortical progenitors and delaying their differentiation into neurons—a plausible mechanism for increasing neuronal output during fetal cortical neurogenesis.^2^^,^^3^ However, partial *NOTCH2* duplications are also present in nonhuman great apes (Figure 1). This leaves open questions regarding (1) when each paralog actually arose, (2) whether the underlying duplications occurred independently in different lineages, and (3) whether the paralogs differ in function, regulation, or patterns of variation within present-day humans or between humans and nonhuman apes. These questions have been difficult to address because the region is large, repetitive, and nearly identical across copies—an arena in which short-read sequencing struggles.

In this issue, Real and colleagues^4^ address these questions by combining near-complete long-read assemblies of human and nonhuman ape genomes,^5^^,^^6^ long-read transcript sequencing, and Fiber-seq—a long-read method that resolves chromatin accessibility at the level of individual DNA molecules and can therefore disentangle nearly identical paralogs.^7^

To reconstruct the duplication history of the *NOTCH2NL* family, the authors compared 12 nonhuman ape haploid assemblies with the human T2T reference and built a maximum-likelihood phylogeny from alignments of *NOTCH2NL* intronic sequences across human, chimpanzee, bonobo, gorilla, and orangutan. The human paralogs form a monophyletic clade to the exclusion of all nonhuman ape sequences, with parallel lineage-specific expansions in gorilla and in the chimpanzee-bonobo branch—a topology most parsimoniously explained by recurrent, independent duplication (Figure 1). Reinforcing this picture, none of the nonhuman ape *NOTCH2* duplications carry the four-base-pair deletion in the terminal exon that is required for stable protein production in human paralogs^2^^,^^4^; copies predicted to encode a stable protein are therefore confined to humans (Figure 1). Molecular dating placed the diversification of these protein-coding human copies at roughly 2.2 to 3.7 million years ago. This broadly coincides with the first documented increases in hominin endocranial volume^8^ and parallels the trajectories of other human-specific duplicated gene families implicated in cortical expansion. Nevertheless, interlocus gene conversion (IGC) between paralogs—the unidirectional transfer of genetic material between homologous sequences—can affect the reliability of these time estimates and thus of conclusions relating to the lineage specificity of these events.

To further elucidate the functional roles of the *NOTCH2NL* paralogs, the authors go on to investigate genetic variation and potential IGC events within this gene family in present-day humans. To this end, they leveraged 70 human haploid genome assemblies from diverse populations and developed a tripartite workflow based on both coding and noncoding sequence similarity among paralogs, as well as on similarity between the genomic regions flanking them. Disagreements between these three assignments were used to flag IGC events,^9^ which proved pervasive, affecting roughly 28% of haplotypes and giving rise to eleven distinct structural configurations (Figure 1). *NOTCH2* and *NOTCH2NLA* were the only paralogs present in every haplotype examined, hinting at a mechanistic or selective bias in conversion direction. The same workflow uncovered a previously undescribed version of *NOTCH2NLR*, termed *NOTCH2tv* (truncated version), in which a tract of ancestral *NOTCH2* sequence has overwritten part of the *NOTCH2NLR* pseudogene. Despite acquiring a *NOTCH2*-like promoter and N terminus, *NOTCH2tv* fails to produce a stable protein in cell-based assays—illustrating that IGC can remodel duplicated sequences but not necessarily resurrect them as functional genes.

To study the regulation of these paralogs, the authors generated Fiber-seq and full-length transcript data from dorsal forebrain organoids derived from a heterozygote individual carrying both the *NOTCH2tv* and *NOTCH2NLR* versions, allowing all paralogs to be compared within a single genomic background. Most accessible regulatory elements within several hundred kilobases of *NOTCH2NL* were shared across paralogs, but their degree of accessibility frequently differed, and a meaningful minority—roughly one in eight—were paralog specific. These paralog-specific elements were concentrated around the two paralogs present in all human haplotypes, *NOTCH2* and *NOTCH2NLA* (Figure 1). Predicted three-dimensional genome organization mirrored this pattern, with each paralog occupying a distinct local environment despite the high underlying sequence identity—echoing observations that human-accelerated regions tend to be associated with rewired topological domains and altered chromatin contacts.^10^

Taken together, the work casts *NOTCH2NL* as a vivid illustration of a broader theme: unstable, duplication-prone genomic regions can serve as engines of adaptive innovation while simultaneously predisposing to disease—the same segmental duplications implicated in cortical expansion also underlie 1q21.1 distal duplication/deletion and neuronal intranuclear inclusion disease, which are characterized by neurodevelopmental abnormalities and chronic neurodegeneration, respectively.

This study skillfully delineates the genetic architecture at the *NOTCH2NL* loci within great apes and humans and establishes a framework for studying some of the most complex evolutionary dynamics in our genome. However, one important caveat is that IGC itself blurs duplication timing, since the divergence being measured may reflect the last conversion event rather than the original duplication, and equally complicates inference of independent versus shared duplication across ape lineages.

Lastly, several intriguing questions remain open. Specifically, we are yet to uncover the selective forces that shaped the apparent fixation of *NOTCH2NLA,* how the paralog-specific accessible chromatin regions affect regulation, and the extent to which these paralogs perform genuinely distinct functions. Notwithstanding, the work provides a rare and comprehensive view of a locus that sits at the intersection of human evolution and disease, opening the door to systematic investigation of duplicated genomic regions that have long remained intractable.

## Declaration of interests

The authors declare no competing interests.

## Declaration of generative AI and AI-assisted technologies in the writing process

During the preparation of this work, the authors used Claude (Opus 4.6, Anthropic) in order to aid in the editing and refinement of the manuscript text. After using this tool, the authors reviewed and edited the content as needed and take full responsibility for the content of the published article.
